# Oncolytic virus and tumor-associated macrophage interactions in cancer immunotherapy

**DOI:** 10.1007/s10238-024-01443-8

**Published:** 2024-08-28

**Authors:** Marc Lecoultre, Paul R. Walker, Aya El Helali

**Affiliations:** 1 Department of Clinical Oncology, Li Ka Shing Faculty of Medicine, Hong Kong University, Hong Kong, China; 2 Division of General Internal Medicine, Geneva University Hospital, Geneva, Switzerland; 3 Faculty of Medicine, University of Geneva, Geneva, Switzerland; 4 Immunobiology of Brain Tumours Laboratory, Center for Translational Research in Onco-Hematology, University of Geneva, Geneva, Switzerland

**Keywords:** Oncolytic viruses, Tumor-associated macrophages, Cancer immunotherapy, Phagocytosis

## Abstract

Oncolytic viruses (OV) are a promising strategy in cancer immunotherapy. Their capacity to promote anti-tumoral immunity locally raises hope that cancers unresponsive to current immunotherapy approaches could be tackled more efficiently. In this context, tumor-associated macrophages (TAM) must be considered because of their pivotal role in cancer immunity. Even though TAM tend to inhibit anti-tumoral responses, their ability to secrete pro-inflammatory cytokines and phagocytose cancer cells can be harnessed to promote therapeutic cancer immunity. OVs have the potential to promote TAM pro-inflammatory functions that favor anti-tumoral immunity. But in parallel, TAM pro-inflammatory functions induce OV clearance in the tumor, thereby limiting OV efficacy and highlighting that the interaction between OV and TAM is a double edge sword. Moreover, engineered OVs were recently developed to modulate specific TAM functions such as phagocytic activity. The potential of circulating monocytes to deliver OV into the tumor after intravenous administration is also emerging. In this review, we will present the interaction between OV and TAM, the potential of engineered OV to modulate specific TAM functions, and the promising role of circulating monocytes in OV delivery to the tumor.

## Introduction

Oncolytic viruses (OV) are a promising strategy to improve immune response against cancer. OV mediates antitumor activity through two mechanisms: selective replication within neoplastic cells, resulting in cell lysis, and the induction of systemic antitumor immunity. They induce immunogenic cell death, release of neoantigens, and the secretion of pro-inflammatory cytokines in the tumor microenvironment (TME). These potentiate tumor-specific immunity [1] with CD8 T cell activation [2]. OVs are selected from native viral species based on their ability to induce immunogenic cell death in cancer cells or engineered to enhance tumor selectivity and increase immunogenicity. Some OVs are already approved for clinical use, such as Talimogene laherparepvec (T-VEC) for melanoma, a genetically modified granulocyte–macrophage colony-stimulating factor (GM-CSF)-expressing herpes simplex virus (HSV). Clinical trials showed that intra-tumoral injection of T-VEC in metastatic melanoma promoted T-cell infiltration and improved the efficacy of immune checkpoint blockade (ICB) with anti-PD-1 and anti-CTLA-4 [3, 4]. In addition to T-VEC, other OV are used in the clinic, such as H101 adenovirus for nasopharyngeal carcinoma, Teserpaturev HSV-1 for recurrent glioblastoma and Nadofaragene firadenovec adenovirus for BCG-unresponsive non-muscle invasive bladder cancer [5].

Furthermore, some challenges remain in OV delivering. Most of the preclinical evidence of efficacy was seen with direct injection of OV into tumors. This may have limitations for disseminated cancer or inaccessible tumors. Therefore, intravenous administration of OV is being explored. However, hemagglutination, the presence of neutralizing antibodies in the blood [1] and sequestration by the mononuclear phagocytic system in the spleen and the liver [6] can result in premature viral clearance and reduced delivery to the tumor site.

Tumor-associated macrophages (TAM) are abundant immune cells in many solid tumors [7]. They derive from monocytes attracted by chemokines such as CCL2 and CSF-1 or tissue-resident macrophages (e.g., microglia in the brain, Kupffer cells in the liver) [8]. Macrophages contain the machinery to build an efficient immune response against cancer cells through their phagocytic activity, antigen presentation, and the secretion of pro-inflammatory cytokines. However, the macrophage phenotype is characterized by a huge plasticity, which makes these cells highly sensitive to their environment. In this context, it was shown that the different parameters that characterize the TME, such as low glucose [9], low oxygen concentration [10], lactic acid [11] and anti-inflammatory cytokines secreted by cancer cells [12] can skew TAM to a more anti-inflammatory phenotype. TAM then secrete the pro-angiogenic factor vascular endothelial growth factor (VEGF-A) that promotes tumor angiogenesis [13], as well as remodeling the TME through the expression of proteases such as matrix metalloproteases and cathepsins that facilitate cancer cell infiltration into surrounding tissue [14]. They also secrete the anti-inflammatory molecules IL-10 [15] and TGFβ [16] and express the immune checkpoint ligand PD-L1 [17] that inhibits T-cell responses. Therefore, the accumulation of TAM in melanoma, lung, colorectal, ovarian, pancreatic, stomach, and kidney cancer is associated with a poorer prognosis [7]. In the literature, TAM phenotype is often referred to as M1 for pro-inflammatory and M2 for anti-inflammatory. M1 and M2 phenotypes were originally obtained in vitro with lipopolysaccharides associated with IFNγ [18] and IL-4 [19] associated with IL-13 [20], respectively. These in vitro characteristics were then extrapolated to TAM in vivo. However, it is now accepted that the TAM phenotype is a continuum in which M1 and M2 represent the two extremes [21]. In the subsequent discussion, we will refer to pro and anti-inflammatory gene signatures of macrophages in vivo and M1/M2 macrophages only when these were polarized in vitro.

OV is a promising approach that harnesses the immune system to build an anti-tumoral response in which TAM plays an important role. This review will discuss the interactions between OV and TAM in cancer immunity. We will first summarize the key roles of macrophages in antiviral response. We will then address how OVs impact TAM function and how TAM impacts OVs efficacy. Finally, we will discuss the exciting field of engineered OV, their capacity to modulate specific TAM functions, and the role of macrophages in delivering OV to the tumor bed, thereby circumventing the limitations of intravenous administration.

## Macrophage responses to viral infection

**Fig. 1:**
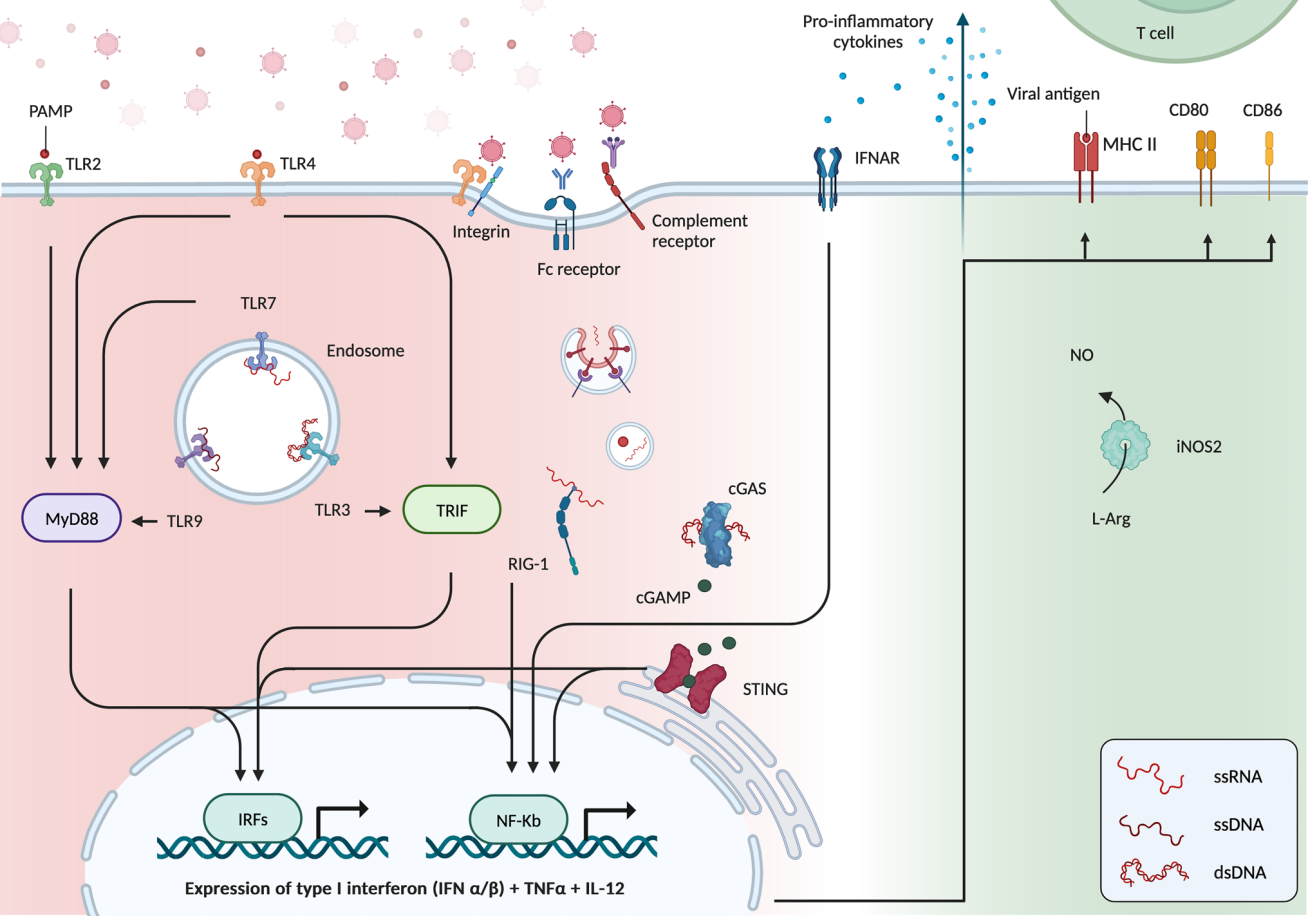
Viral recognition by macrophages (left) and macrophage activation (right). Different PRRs, including TLR2 and TLR4, recognize PAMP on the macrophage surface. Their downstream activation leads to the expression of the pro-inflammatory cytokines type I interferons, TNFα and IL-12. In association with other receptors, such as integrins, PRR can induce virus endocytosis. In the endosome, TLR3 recognizes dsRNA, TLR7 recognizes ssRNA, and TLR9 recognizes viral DNA. Viruses opsonized with either antibodies or complements are recognized by Fc receptors and complement receptors, respectively. These receptors do not directly induce pro-inflammatory molecule secretion but initiate virus phagocytosis. Once in the phagosome, viral elements are digested, but PRR can still recognize PAMP and viral antigens can be recruited to present on MHC-II to CD4 T cells. In the cytosol, free RNA and free DNA are recognized by RIG-1 and cGAMP, respectively. Once activated, other than the secretion of the pro-inflammatory cytokines mentioned above, macrophages secrete NO, increase MHC-II expression for antigen presentation, and increase the expression of the costimulatory molecules CD80 and CD86. cGAMP = cyclic guanosine monophosphate-adenosine monophosphate; cGAS = cyclic GMP-AMP synthetase; dsRNA = double stranded RNA; IFN = interferon; IFNAR = IFNA receptor; IRF = interferon regulator factor; MyD88 = myeloid differentiation primary response protein 88; PAMP = pathogen-associated molecular patterns; PRR = pattern recognition receptors; RIG-1 = retinoic acid-inducible gene I; ssRNA = single stranded RNA; STING = stimulator of interferon genes; TLR = Toll-like receptor; TRIF = Toll/IL-1R domain-containing adaptor-inducing IFNβ

Macrophage activation by viruses requires their detection, followed by the internalization of viral material (Fig. 1). Macrophages recognize viral conserved molecular structures known as pathogen-associated molecular patterns (PAMP). They are unique to viruses and are not produced by host cells. Different types of molecular structures can act as viral PAMP, such as viral envelope glycoproteins, double stranded RNA (dsRNA), single stranded RNA (ssRNA), DNA, CpG DNA, and nucleic acid motifs [22]. On the macrophage surface, PAMP is recognized by pattern recognition receptors (PRR), the most widely described being Toll-like receptors (TLR)-2 and 4, which specifically sense viral envelope glycoproteins [23]. Many PRRs have been described and recognize different viral PAMP [24]. Notably, different PRRs are involved in DNA and RNA virus recognition. Not every PRR can induce viral engulfment on its own and, therefore, requires the interaction between the virus and other receptors, such as integrins or gangliosides [25]. The recognition of viral elements initiates signaling pathways that trigger membrane remodeling and the formation of a nascent vacuole that encloses the virus and moves it inside the cytoplasm [26]. Viral uptake is mediated either by endocytosis or by phagocytosis, depending on the receptor involved at the macrophage surface [27]. Because of their small size, viruses are generally engulfed by endocytosis. However, viral opsonization by neutralizing antibodies or complement, recognized by macrophages through Fc receptors or complement receptors 1 and 3, respectively [28] induces phagocytosis [27]. Macrophages can also phagocytose infected cells and recognize viral elements after processing in the phagosome [29].

Within macrophages, viral PAMP is recognized in the endosomes/phagosomes and directly in the cytoplasm. The main PRRs are TLR3, TLR7, and TLR9 in endosomes and retinoic acid-inducible gene I (RIG-1), cyclic GMP-AMP synthetase (cGAS), and RNA-dependent protein kinase (PKR) in the cytoplasm. All endosomal TLRs sense nucleic acids but have different downstream signals [30, 31]. TLR3 signals through Toll/IL-1R domain-containing adaptor-inducing IFNβ (TRIF) that activates the transcription factors IRF3 and IRF5. TLR7 and TLR9 signal via the myeloid differentiation primary response protein 88 (MYD88), which activates the transcription factors NF-Kβ, IRF3, and IRF7. The downstream effect is an up-regulation of MHC-II expression on the cell surface and secretion of the pro-inflammatory cytokines IL-12 and type I interferons INFα and IFNβ [32]. RIG-1 recognizes viral RNA in the cytoplasm, and its downstream activation also leads to IFNβ expression [33]. Type I interferons (INFα and IFNβ) are key players in the antiviral response. In cancer cells, they induce cell cycle arrest, promote apoptosis, and inhibit protein synthesis [6]. In the TME, they provide antiangiogenic signals and potentiate CD4 and CD8 T-cell activation to promote an antigen-specific response [34]. Macrophages sustain activation in an autocrine manner by binding to IFN receptors on the cell surface [35]. In addition to RIG-1, cytoplasmic cGAS recognizes viral DNA and produces cGAMP that complexes with STING, which, through IRF3 and NF-κB induces the secretion of type I interferons and TNF-α [36]. Another important cytoplasmic player is PKR, which, by recognizing double-stranded RNA, is a critical factor in clearing intracellular viral infections [37].

Once activated, macrophages secrete reactive oxygen species and nitric oxide (NO) that suppress viral replication by impairing viral ribonucleotide reductase activity [38]. NO is synthetized by the enzyme iNOS2, whose expression level is used as a pro-inflammatory marker of macrophages. Conversely, macrophages can participate in adaptative immunity by presenting intracytoplasmic antigens on MHC-I to CD8 T cells and phagocytosed antigens on MHC-II to CD4 T cells. Expression of the costimulatory molecules CD80 and CD86 increases, participating in T-cell activation through CD28 binding [39]. Taken together, antigen presentation, costimulatory molecule expression, and secretion of type I interferons and other pro-inflammatory molecules contribute to robust T-cell response against viral antigens. Macrophages play a key role in antiviral response. However, it is important to emphasize that another immune cell population that is important in this process is dendritic cells (DC). Indeed, plasmacytoid DCs are the main sources of type I IFN after viral infection, and other DC populations are the principal cells responsible for initiating T-cell responses [40, 41].

Viruses also infect parenchymal cells where some of the mechanisms described above, such as TLR and cGAS, are present. However, regarding cancer cells, some elements of the antiviral machinery, such as PKR [42] or STING signaling [43] are lost. For this reason, the induction of immunity may be limited, and viral maintenance can be more easily tolerated in cancer cells, a feature that OV exploits to increase their penetrance in tumors. This potentially impacts TAMs as reduced type I interferon secretion by infected cancer cells will limit the induction of TAM pro-inflammatory functions.

Overall, the mechanisms described above induce a pro-inflammatory activation state of macrophages. However, some nuances regarding the viral species and the infection stage must be highlighted. Indeed, some viruses have developed mechanisms to circumvent macrophage antiviral activity for efficient replication. HSV, one of the most commonly used OV in preclinical models, can produce the protein ICP27 that interferes with the cGAS-STING signalosome, thereby inhibiting type I IFN production in human macrophages [44]. In a model of the infected cornea, HSV up-regulated the expression of PD-L1, a key immune checkpoint involved in T cells and macrophage inhibition [45]. Myxoma virus, explored as an OV in preclinical models, can encode a homolog of CD200 that binds to CD200R on macrophages and inhibits their M1 polarization in vitro [46]. Finally, the vaccinia virus, also used as an OV, inhibited NO production by macrophages and induced their apoptosis [47]. Detailed mechanisms of viral resistance to macrophage activation are beyond the scope of this review, and we refer the reader to other publications that address this subject more comprehensively [48–50]. However, it highlights the importance of understanding virus biology in manufacturing efficient OV.

## OV impact on TAM

### Polarization

TAM polarization between pro and anti-inflammatory status impacts the responses of other immune cells and reshapes the TME. Specific gene signatures are used to define each extreme of the continuum. TAM are considered anti-tumoral (M1) when they express TNFα, CD80, CD86, IL-1, IL-12, iNOS or MHC-II and pro-tumoral (M2) when they express Arg1, Mrc1 or CD163 [12].

In two medulloblastoma syngeneic models, Hedberg et al. showed with single-cell analysis that TAM abundance was increased upon treatment with HSV-C134 and that they expressed more of the pro-inflammatory marker STAT1, a transcription factor involved in interferon expression. The positive impact of OVs on survival was preserved in athymic nude mice, showing that the anti-tumoral effect was independent of T cells [51]. Similar findings regarding TAM phenotype and T-cell independent tumor growth inhibition were observed in different subcutaneous human gastric cancer xenograft models treated with HSV G47Δ [52] and in a subcutaneous human uveal melanoma xenograft model treated with HSV-1 [53].

**Fig. 2:**
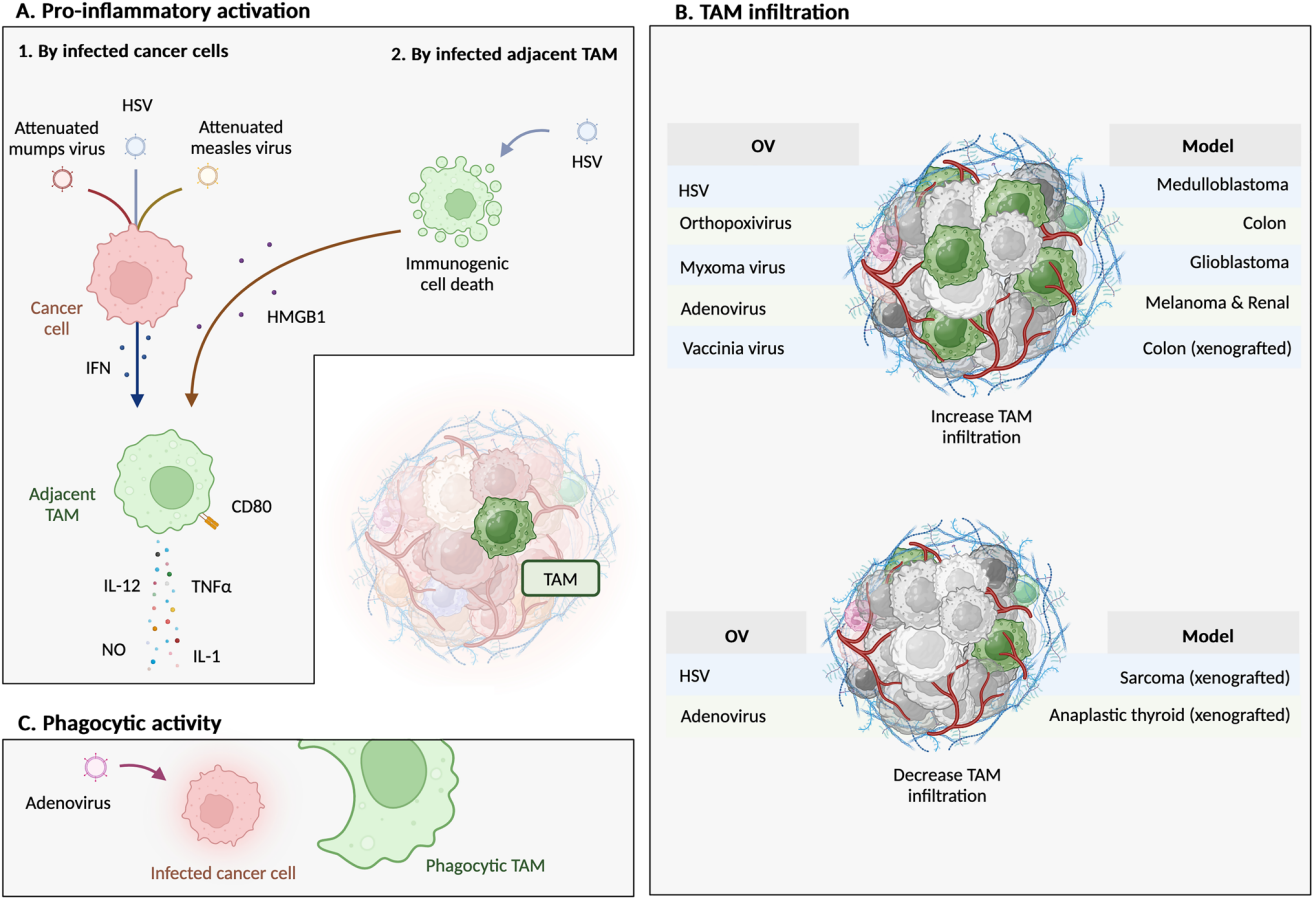
OV impact on TAM. **A**. By infecting cancer cells, some OV induce a pro-inflammatory activation of surrounding TAM (with pro-inflammatory cytokines secretion and CD80 expression). In addition, by infecting TAM, HSV induces immunogenic cell death with HMBG1 secretion, which has the potential to activate non-infected adjacent TAM. **B**. Some OVs increase TAM infiltration, and others decrease it. Here is showed this OV impact in which model. **C**. Cancer cells infected by an adenovirus are more easily phagocytosed

In human cells, monocyte-derived macrophages increased expression of the costimulatory molecule CD80 and secretion of the pro-inflammatory cytokines IL-1 and TNFα when they were exposed to conditioned media of the breast adenocarcinoma cell line MDA-MB-231 infected with attenuated mumps or measles viruses (Fig. 2A). Similar modifications were observed when macrophages were directly infected. This confirms that viral infection of macrophages or surrounding cancer cells can promote pro-inflammatory functions [54]. Using the PyMT-TS1 breast cancer model, Kwan et al. showed that TAM expressed higher levels of the pro-inflammatory genes IL-12 and iNOS when the mice were treated with HSV-1716 [55]. In vitro, human monocyte-derived macrophages supported HSV-1716 replication, leading to immunogenic cell lysis with HMGB1 secretion. This interesting finding suggests that in parallel to immunogenic cell death of tumor cells, a similar process can occur in infected TAM, which could potentiate the pro-inflammatory functions of adjacent non-infected TAM. In line with this, the antitumor effect of OV was diminished when TAM infiltration was decreased using liposomal clodronate (a small molecule taken up specifically by phagocytes due to its liposomal formulation and that kills the phagocytes through inhibition of the mitochondrial electron transport chain) [55].

Regarding the evidence in patients, Miller et al. performed bulk sequencing of six recurrent glioblastomas between two and five days after the intra-tumoral injection of HSV-G207. Compared to tumors pre-treatment, the TME showed more pro-inflammatory gene expression and more macrophage infiltration, but the whole tissue sequencing did not permit specific characterization of the macrophage transcriptome [56]. Another study in 15 recurrent glioblastoma patients showed that TNFα, IFNγ and IL-6 were increased in cerebrospinal fluid 72 h after treatment with the adenovirus Delta24-RGD [57]. Nevertheless, since the anti-inflammatory cytokine IL-10 was also increased, this highlights the complexity of the immune response induced after oncolytic virus therapy.

Besides the native virus effect described above, some oncolytic viruses have been specifically engineered to encode pro-inflammatory cytokines, such as T-VEC, that encode GM-CSF. Similarly, in mouse models, B16-F10 melanoma infected by an adeno-associated virus encoding GM-CSF increased MHC-II expression and TNFα, IL-6 and IL-12 secretion by macrophages [58]. A vesicular stomatitis virus engineered to encode IFNσ increased CD86 expression on CD11b^+^ tissue-resident spleen macrophages after intravenous administration in mice [59]. CD86 increase was also observed when parental vesicular stomatitis virus was injected, suggesting that the virus induced pro-inflammatory macrophage activation. Parental vesicular stomatitis virus decreased lung metastasis occurrence in the 4T1 mammary adenocarcinoma model but was more efficient when engineered to secrete IFNσ.

Overall, oncolytic viruses induce a pro-inflammatory phenotype of macrophages. This was described mainly with HSV and adenoviruses in different cancer models. To facilitate intra-tumoral injection of OV, most of the models used were subcutaneous tumors. As the subcutaneous microenvironment may differ from other organs, the observations we described above must be considered in light of this parameter. The limited evidence in tumors from patients shows a more pro-inflammatory TME, but polarization or function of TAM has not been specifically addressed. As antiviral mechanisms in some human cancer cell lines are defective [60], it can be speculated that their ability to secrete type I interferons is reduced and, therefore, might be insufficient to induce TAM anti-inflammatory functions. Therefore, the interesting finding is that infected TAM can undergo immunogenic cell death [55]. However, confirmation of direct TAM infection by OV in patient samples has rarely been reported. For instance, Ramelyte et al. monitored T-VEC response in 13 patients with primary cutaneous B cell lymphoma by repeated fine-needle aspirates of injected and non-injected sites [61]. Single-cell transcriptomic analysis revealed that macrophages contained T-VEC transcripts. The authors suggest that in addition to specific cancer cell infection, which is the proposed anti-tumoral mechanism of T-VEC [62], it also infected TAM, promoting their anti-tumoral response. However, the exact viral uptake mechanism (viral infection vs phagocytosis of viral elements or infected cancer cells) is unknown.

### Infiltration

TAM infiltration in tumors is generally associated with a poorer prognosis [7]. However, as OV potentially reshapes the TAM function, an increased abundance may benefit the anti-tumoral immune response.

TAM infiltration increased in the syngeneic medulloblastoma models [51] mentioned above. TAM abundance was increased in the colon cancer model MC38 two days after injecting orthopoxvirus-CF33 [63] (Fig. 2B). However, this effect was lost after ten days. OV derived from adenoviruses increased TAM in melanoma, renal adenocarcinoma [64], and glioblastoma model [65]. In the latter study, F4/80^+^ TAM abundance was quantified by immunohistochemistry, which may have limitations due to spatial heterogeneity of TAM distribution. Myxoma virus increased myeloid cells in a glioblastoma model, but here, no distinction was made between monocyte-derived macrophages and neutrophils [66]. Finally, TAM abundance was increased in a subcutaneous human xenograft colon cancer model treated with vaccinia virus GLV-1h68 [67]. However, in these xenograft models, immune cell infiltration should be interpreted cautiously, as the myeloid compartment is aberrant. Indeed, the mixture of mouse and human cytokines from tumor cells with varying degrees of cross-reactivity on mouse cells constitutes a microenvironment different from that found in syngeneic models. Regarding the underlying mechanism of TAM abundance increase, Lee et al. showed that human glioblastoma and breast cancer cells infected with HSV-1 increased the expression of the monocyte chemo-attractant CCL2 [68].

In contrast, HSV in a sarcoma xenograft model decreased TAM infiltration. The concomitant increase of neutrophil infiltration in the tumor may be responsible for this relative decrease [69]. Passaro et al. demonstrated the potential of adenovirus-dl922-947 to decrease TAM infiltration in an anaplastic thyroid cancer model, despite the limitations of the immunohistochemistry technique. In vitro, CCL2 secretion by infected cancer cells was decreased, which consequently limited monocyte chemotaxis [70].

Overall, abundant evidence for increased TAM infiltration upon OV treatment exists, but in some cases, decreased TAM infiltration was reported in xenograft models. Further research is needed to confirm that OVs decrease TAM infiltration in specific tumors such as sarcoma and anaplastic thyroid cancer and that this impact is reproducible in syngeneic models. It is also probable that different species and strains of viruses have different capacities to attract monocyte-derived macrophages, but this question has not been specifically addressed yet.

### Phagocytosis

Phagocytosis of cancer cells by TAM plays an important role in cancer immunity. Macrophages interact with their target cells through eat-me receptors and eat-me signals promoting phagocytosis [71]. In parallel, don't eat me signals can be expressed by cancer cells and recognized by don't eat me receptors on macrophages. The balance between eat-me and don't eat me signals on the cancer cell surface dictates the induction of phagocytosis. It has been shown that the increase of phagocytic activity through the don't eat me signal CD47 inhibition showed promising results in many preclinical models [72, 73]. In addition, phagocytosed cancer antigens can be presented to CD4 T cells on MHC-II [74]. TAM thereby activates an adaptative antitumor immunity in parallel to their phagocytic activity [75]. However, the presentation of phagocytosed antigens to CD8 T cells on MHC-I through cross-presentation is still controversial [76].

Bolyard et al. [77] used an engineered HSV to interfere with the membrane receptor Brain Angiogenesis Inhibitor 1 (BAI1) expressed by TAM. BAI1 is expressed by microglia and macrophages and is an eat-me receptor recognizing phosphatidyl-serine in apoptotic cells [78]. In addition, it induces a pro-inflammatory response when acting as a PRR recognizing lipopolysaccharides in Gram-negative bacteria [79]. In a human xenograft orthotopic glioma model, the treatment with an HSV expressing the extracellular fragment of BAI1 (Vstat 120) and interfering with its function decreased CD11b^+^ myeloid cell infiltration and expression of the pro-inflammatory molecules MHC-II and CD86. In vitro, when bone-marrow derived macrophages from *Bai1*^−/−^ mice were in co-culture with human glioma cells infected with HSV, the expression of TNFα by *Bai1*^−/−^ macrophages was decreased compared to wild-type macrophages, suggesting that BAI1 downstream activation leads to TNFα expression increase. This would imply that it is the pattern recognition activity of BAI1 that was involved in the process rather than the recognition of apoptotic cells. Indeed, recognition of phosphatidyl-serine is expected to induce anti-inflammatory functions and decrease TNFα expression, as shown with other eat-me receptors [80]. Even though BAI1 activity as a PRR has been described for Gram negative bacteria, its involvement in viral element recognition is not excluded.

Regarding eat-me signals expressed by infected cancer cells, HSV-1 expressing GM-CSF infection induced calreticulin expression in different cancer cell lines [81]. Calreticulin is an eat-me signal induced by cellular stress [82]. Whether the virus or GM-CSF was the main inducer of calreticulin expression was not addressed, and no phagocytic activity was quantified. However, this interesting finding suggests that through calreticulin expression induction, HSV-1 infection may facilitate cancer cell phagocytosis by macrophages. Some authors report that specific antibodies could recognize viral antigens on the surface of infected cancer cells and induce antibody-dependent cell phagocytosis [83]. Even though this mechanism is biologically valid, it was described for antibody-dependent cellular cytotoxicity but not for antibody-dependent cell phagocytosis [84].

The research discussed above did not specifically quantify phagocytic activity against cancer cells. This was measured with HSV, which did not increase phagocytic activity against glioblastoma cells in vitro [85] and in vivo [86], nor against ovarian cancer cells in vitro [87]. In contrast, glioblastoma cells infected with an adenovirus underwent higher phagocytosis by *ex-vivo* TAM compared to non-infected glioblastoma cells [88] (Fig. 2C).

Overall, the limited existing evidence shows that HSV does not promote phagocytic activity against infected cells, even though it can increase their calreticulin expression. As phagocytosis induction depends on the balance between many eat-me and don't-eat me signals on the cancer cell surface, the increase of only one of them (for example, calreticulin) may be insufficient to have an impact. In contrast, adenovirus slightly increased phagocytic activity against infected cells.

In addition to TAM phagocytic activity against cancer cells, there is evidence for direct OV uptake in patients. TAM of glioblastoma patients treated with the delta24-RGD adenovirus contained hexon peptides, components of the viral capsid protein [57]. In vitro, M1 macrophages more efficiently engulfed hexon peptides than M2 macrophages. It has been demonstrated that macrophages are resistant to wild-type adenoviral replication [89]. Thus, TAM hexon-positivity is presumably caused by the active engulfment of OVs. However, the exact mechanism involved (endocytosis vs phagocytosis) was not explored.

An important question that has not been addressed to date is whether TAM phagocytic activity leads to antigen presentation to T cells. In a syngeneic murine intracranial melanoma model, intra-tumoral injection of HSV increased the abundance of CD4 T cells and TAM [90], and the effect on survival was dependent on CD4 T cells [91]. However, there was no evidence that TAM mediated CD4 T-cell priming. Similar findings were shown in a mouse model of colon cancer, where the recombinant orthopoxvirus increased infiltration of TAM and activated CD8 T cells [63] but without evidence of antigen presentation by TAM.

## TAM impact on OV efficacy

OV penetrance in tumors and replication in cancer cells are essential for their efficacy. Antiviral immune responses may block virus replication and inhibit infection of tumor cells. Therefore, pro-inflammatory macrophages can potentially hinder OV activity by inducing an antiviral immune response inside the tumor.

In two xenograft models of Ewing sarcoma treated with HVS-rRp450, Denton et al. showed that the TAM infiltration decreased after liposomal clodronate treatment, which enhanced OV antitumor efficacy without increasing virus replication [92]. Using a similar approach in a rat glioma model in which monocyte-derived TAMs were depleted, but brain resident microglia were preserved, HSV-hrR3 efficacy was increased [93]. Interestingly, in a syngeneic mouse glioma model treated with a myxoma virus, Zemp et al. showed that microglia play the main role in viral clearance [66]. While the depletion of only monocyte-derived TAM with clodronate liposome had no impact, the depletion of monocytes and microglia with cyclophosphamide increased the survival of mice treated with myxoma virus. Cyclophosphamide also increased HSV replication in a rat glioma model, associated with decreased TAM infiltration, without distinguishing between monocyte-derived macrophages and microglia [94]. The positive impact of cyclophosphamide on virotherapy is interesting as it is a drug used in different standard chemotherapy regimens and was, therefore, tested in association with OV in other models [95]. However, these preclinical findings have limitations. Indeed, cyclophosphamide effects vary according to its dosage. High doses result in cytotoxicity and innate immunosuppression, but low dose achieves immunostimulatory effects, including suppressive Treg depletion and expansion of antigenic-specific T cells [96]. Moreover, cyclophosphamide induces immunogenic cell death [97], which enhances antitumor immunity. Therefore, the anti-tumoral impact seen in these studies and interpreted as being mediated by TAM depletion could also have been partially mediated by the other effects of cyclophosphamide.

Regarding TAM pro-inflammatory and antiviral impact, in vitro, HSV-1 can infect rat microglial cells, inducing STAT1 phosphorylation and increasing their phagocytic activity against latex microbeads. Consistent with this, rat microglial cells inhibited HSV-1 replication in human glioblastoma cells. In vivo, inhibition of STAT1 with the oxindole/imidazole derivate C16 increased viral load in human glioblastoma xenograft tumors implanted in athymic nude mice and inhibited tumor growth [98]. The role of specific pro-inflammatory cytokines has been investigated. In vitro, both IFNα and conditioned media of macrophages were shown to confer resistance against the oncolytic effect of VSV in murine ovarian cancer cells [99]. It was, therefore, hypothesized that macrophages could secrete IFNα which confers this resistance. It is important to exercise caution while interpreting these results as the macrophage cells utilized in the study belonged to the acute leukemia cell line RAW264. This particular cell line may less accurately reflect the biology of TAM as compared to macrophages derived from bone marrow. Focusing on IFNα secreted by infected cancer cells, it was shown in the RG2 rat glioma cell line treated with the myxoma oncolytic virus that the adjunction of the mTOR inhibitor rapamycin decreased the expression of IFNα in vitro. When the cells were forming tumors in vivo, rapamycin decreased TAM infiltration and increased the therapeutic efficacy of the myxoma virus [100]. This suggests that in this model, TAM exposed to IFNα interferes with the anti-tumoral activity of the myxoma virus. Besides IFNα, TNFα also plays a role in macrophage responses against viruses. Meisen et al. showed that the co-culture of HSV-1 infected human glioblastoma cells with macrophages derived from the bone marrow of TNFα^−/−^ mice increased viral titers. In athymic mice implanted with human glioblastoma cells intracranially, the co-administration of HSV-1 with anti-TNFα increased mouse survival [101].

CCN1 is a secreted protein found within the extracellular matrix that induces pro-inflammatory functions in macrophages by binding to cell-surface integrins [102]. In co-cultures of macrophages and glioblastoma cells infected with HSV-1, CCN1 blockade decreased macrophage migration toward cancer cells and reduced viral clearance [103]. In athymic nude mice implanted subcutaneously with human glioblastoma cells, pre-treatment with anti-CCN1 decreased TAM infiltration in the tumor and increased HSV abundance.

In contrast to the pro-inflammatory cytokines discussed above, anti-inflammatory cytokines such as TGFβ and IL-10 inhibit macrophage antiviral responses. Han et al. showed in vitro that HSV titers increased when infected human glioblastoma cells were co-cultured with mouse macrophages pre-treated with TGFβ [104]. Similar results were observed with the microglial cell line BV2. In an immunocompetent glioblastoma model treated with HSV, the administration of TGFβ decreased TAM infiltration and their TNFα and NOS2 expression [104]. In this study, the definition of TAM populations by flow cytometry did not exclude neutrophils, so it is possible that this population also plays a role. Mice treated with HSV and TGFβ survived longer than those treated with HSV only, but the role of NK cells seemed predominant [104]. In the MC38 peritoneal carcinomatosis model treated with vaccinia virus, Guo et al. showed that the immunosuppressive cocktail mycophenolic acid, dexamethasone, and tacrolimus increased TAM infiltration and their expression of IL-10 compared to control peritoneal macrophages. Mice treated with the immunosuppressive cocktail and vaccinia virus showed longer survival than those treated only with the virus [105]. This suggests that the balance between the oncolytic activity of the vaccinia virus was superior to the anti-inflammatory functions of macrophages on anti-tumoral responses.

The capacity of macrophages to recognize viruses can also be exploited. TLR on the macrophage surface recognize glycosylated proteins. Therefore, deglycosylating the viral surface proteins can inhibit virus recognition. This approach was tested with vaccinia virus, which showed increased viral penetrance in subcutaneous tumors [106]. However, the role of macrophage vs cancer cell TLR was not addressed. Even if this approach is elegant, its main limitation is that after the first cycle of replication, the virus uses the infected cell membrane to constitute its envelope, and the surface viral proteins are glycosylated again.

In addition, macrophages can clear OV in the periphery and decrease OV bioavailability in intravenous administration. For instance, it was shown that PI3Kδ inhibition decreases vaccinia virus uptake by spleen macrophages, thereby increasing viral delivery to subcutaneous tumors [107].

Overall, strong evidence in different cancer models shows that pro-inflammatory TAM can have a detrimental effect on OV efficacy, mainly by decreasing viral persistence in tumors. In this context, some authors suggested that OV could be combined with immunosuppressive treatment to increase efficacy [6]. This approach is debatable, as the survival advantage was lost in models where OV efficacy was T-cell dependent [65]. Indeed, some of the results discussed above were observed in xenograft models lacking adaptative immunity. Therefore, the role of innate immunity in the balance between antiviral and anti-tumoral immunity may have been overestimated. Further research is needed to explore the balance of macrophage immunity against viruses and cancer. If key differences could be identified and harnessed, this would support an approach by which TAMs are specifically modulated to have anti-tumoral functions while being tolerant of OV.

## Engineered OV

Promising research has modified OVs in different ways to build a more robust cancer immunity. We already mentioned some of them, such as an adenovirus secreting GM-CSF [58], a vesicular stomatitis virus engineered to encode IFNσ [59], and an HSV secreting Vstat 120 to inhibit the PRR BAI [77]. Here, we will discuss other approaches that impacted TAM or were designed to harness specific TAM functions.

**Fig. 3:**
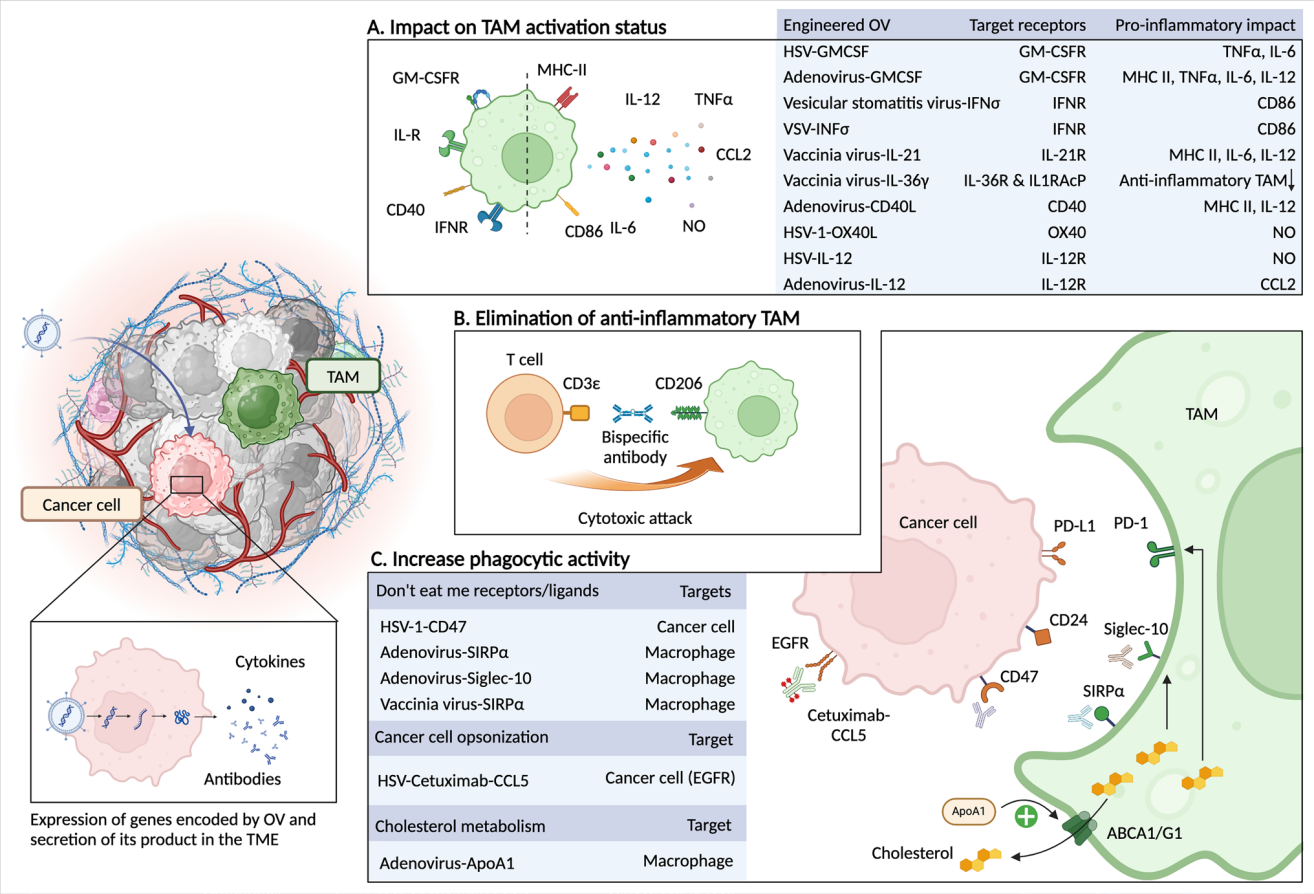
Mechanisms potentially used by engineered OV to modulate TAM functions. Engineered OVs infect cancer cells that enable the expression of the encoded genes such as cytokines, antibodies, or ApoA1. **A.** OV engineered to secrete pro-inflammatory cytokines induces a pro-inflammatory activation status of macrophages, with an increased expression of MHC-II and CD86 and increased secretion of IL-6, IL-12, TNFα and NO. **B**. OV engineered to secrete a bispecific antibody targeting CD3ε and CD206 induces the depletion of anti-inflammatory TAM by T cells. **C**. OV engineered to secrete antibodies targeting the don't eat-me ligand SIRPα and Siglec-10 or the don't eat me receptor CD47 increase TAM phagocytic activity. The secretion of ApoA1 activates ABCA1/G1 on macrophages, which decreases their intra-cellular cholesterol concentration. Because high cholesterol concentration in macrophages favors the expression of the don't eat me ligands Siglec-10 and PD-1, decreased cholesterol promotes TAM phagocytic activity. Cetuximab linked to CCL5 increases TAM infiltration and phagocytic activity. ABCA1/G1 = ATP-binding cassette subfamily A member 1 G1; ApoA1 = Apolipoprotein A1; EGFR = Epithelial growth factor receptor; NO = nitric oxide; VSV = vesicular stomatitis virus

One approach of OV engineering has been to promote pro-inflammatory cytokine secretion by TAM. Eriksson et al. engineered an adenovirus armed with a trimerized membrane-bound extracellular CD40L, enabling infected cells to express CD40L (the ligand of CD40) on their surface [108]. CD40 is an interesting target in cancer immunotherapy due to its ability to stimulate T-helper 1 immunity via the maturation of dendritic cells and to drive pro-inflammatory macrophage activation [109]. In vitro, human monocytes exposed to pancreatic cancer cells infected with the engineered adenovirus increased MHC-II and IL-12 expression [108] (Fig. 3A). Marelli et al. engineered a vaccinia virus to secrete IL-21 (VV-IL-21) [110]. IL-21 is a pro-inflammatory cytokine that induces T cell activation [111], inhibits the development of Treg [112], and induces maturation and activation of NK cells [113]. In vitro, macrophages in contact with VV-IL-21 infected pancreatic cancer cells showed increased expression of IL-6 and IL-12. In a mouse syngeneic pancreatic cancer model, TAM increased expression of MHC-II when treated with VV-IL-21, in contrast to tumors treated with parental vaccinia virus in which TAM MHC-II expression was unchanged. OV-IL-21 showed antitumor efficacy, which was partially CD8 T-cell mediated [110]. IL-36γ activates DC, T cells, and NK cells and is believed to function as an alarmin in damaged tissues [114]. IL-36γ armed vaccinia virus induced stronger anti-tumoral activity in MC38 colon cancer, Panc02 pancreatic cancer, and B16 melanoma models. In MC38, infiltration of F4/80^+^ CD206^+^ anti-inflammatory TAM decreased upon treatment with the OV [115]. Others targeted OX40, which belongs to the tumor necrosis factor receptor superfamily and is expressed on activated T cells. It acts as a costimulatory signal when it binds to its ligand OX40L [116]. Liu et al. engineered an HSV-1 expressing OX40L, which showed in the pancreas cancer model KPC implanted subcutaneously an increase of iNOS expression by TAM but without statistical significance [117]. Synergy was noted between HSV-1 expressing OX40L and anti-IL-6 on tumor growth inhibition. Finally, harnessing the pro-inflammatory propriety of IL-12, Saha et al. could cure most mice implanted with GSC-005 glioblastoma cells when a HSV-G47Δ expressing murine IL-12 was associated with anti-PD-1 and anti-CTLA-4 blockade [118]. When injected alone, the OV did not modify the abundance of TAM, but expression of the pro-inflammatory genes iNOS and STAT1 was increased. A synergistic impact on survival was observed with OV and anti-PD-1/anti-CTLA-4 blockade. The effect was dependent on CD4 and CD8 T cells and on macrophages, as shown when peripheral monocytes were depleted by clodronate liposomes. Interestingly, in association with anti-PD-1 and anti-CTLA-4, OV increased the expression of PD-1 on intra-tumoral CD11b^+^ myeloid cells. As PD-1 has been described to inhibit phagocytic activity of macrophages when bound to PD-L1 expressed on target cells [119], this observation could imply that the association of OV with anti-PD-1/anti-CTLA-4 blockade could decrease GSC-005 TAM phagocytic activity. This is, however, theoretical, as expression of other eat-me or don't eat me receptors on CD11b^+^ cells infiltrating GSC-005 tumors was not measured. Another approach using an adenovirus expressing IL-12 induced also a pro-inflammatory TME in a breast cancer model and an increased CCL2 expression [120]. The origin of CCL2 was not addressed, but as monocytes and macrophages are the main source of this cytokine, it can be hypothesized that adenovirus-IL-12 promotes CCL2 secretion by TAM.

An alternative way to modulate TAM through engineered OV has been to specifically deplete anti-inflammatory macrophages. To this end, Scott et al. engineered an adenovirus to secrete a bispecific T cell engager recognizing CD3ε on T cells and the M2 marker CD206 on macrophages [121] (Fig. 3B). This led to CD8 T-cells cytotoxic activity against CD206^+^ macrophages and IFNγ secretion by CD4 T cells. Treatment with this OV decreased CD206 ^+^ macrophage abundance in ascites and increased CD80 and CD86 expression on the remaining macrophages, potentially induced by IFNγ secreted by T cells.

TAM phagocytic activity is another function targeted by engineered OV. Xu et al. engineered HSV-1 to make infected cells secrete anti-CD47 antibodies [86] (Fig. 3C). In vitro, OV-anti-CD47 increased phagocytic activity of human macrophages against glioblastoma cells through antibody dependent phagocytosis mediated by the Fc portion of the secreted IgG_1_. Expression of the pro-inflammatory cytokines IL-1, IL-6, and IL-12 was increased. In the CT2A immunocompetent glioblastoma model, OV-anti-CD47 increased TAM phagocytic activity and increased survival compared to mice treated with the native virus. Similar findings were observed in the ID8 immunocompetent ovarian model [87]. The synergy between OV and anti-PD-L1 was observed, but the exact mechanism of anti-PD-L1 (activating T cells or increasing phagocytic activity) was not addressed [87]. Xie et al. engineered an adenovirus to secrete SIRPα-Fc, Siglec-10-Fc or TIGIT-Fc, binding to CD47, CD24, and CD155, respectively [122]. Inhibition of SIRPα and Siglec-10, two don't eat me receptors, significantly suppressed tumor growth of the MC38 colon cancer model. Surprisingly, OV-SIRPα compared to native adenovirus decreased the relative proportion of TAM, which may be due to the parallel massive increase of CD8 T-cell infiltration. The phagosome pathway was enriched in TAM, but no direct quantification of phagocytic activity was performed. A vaccinia virus was able to disrupt CD47/SIRPα interaction by secreting the chimeric molecule ectodomain SIRPα and the Fc domain of IgG_4_ [123]. In vitro, M1 and M2 macrophages killed more efficiently infected tumor cells, but phagocytic activity was not tested. In an immunocompetent osteosarcoma model, the engineered vaccinia virus increased CD11b^+^ infiltration compared to non-treated tumors and increased mice survival compared to those treated with the native virus [123]. HSV was modified to express a secretable single-chain variable fragment of the epidermal growth factor receptor (EGFR) specific antibody cetuximab linked to CCL5 by an Fc knob-into-hole strategy [85]. This fusion protein could bind to wild-type EGFR and mutant EGFR-vIII on tumor cells. CCL5 is an inflammatory chemokine that promotes the chemotaxis of immune cells by interacting with CCR1 and/or CCR5 [124]. In vitro, OV-Cmab-hCCL5-infected human glioblastoma cells supernatant increased the phagocytic activity and IL-1, IL-6, IL-12, and NOS2 expression of human monocyte-derived macrophages [85]. In the immunocompetent glioblastoma model CT2A, OV-Cmab-hCCL5 increased macrophage infiltration and survival. The impact on survival was partially abolished after monocyte-derived macrophage depletion, suggesting that persisting microglia may also be involved in the immune response. Finally, cholesterol metabolism was targeted to favor phagocytic activity. Disrupted cholesterol homeostasis is crucial in maintaining an immunosuppressive TME [125]. Wang et al. showed that abnormal accumulation of cholesterol in glioblastoma monocyte-derived TAM induced up-regulation of ABCA1/G1 cholesterol efflux receptors and don't eat-me receptors, which led to TAM with impaired phagocytic activity [88]. In vitro, the accumulation of cholesterol in bone marrow-derived macrophages decreased their phagocytic activity, and the restoration of cholesterol efflux with ApoA1 treatment increased their phagocytic activity against glioblastoma cells. In a glioblastoma mouse model, the cholesterol level in TAM was higher than in tumor-infiltrating monocytes and peripheral macrophages isolated from the spleen, indicating that TAM accumulated cholesterol. The don't eat me receptors Siglec-10 and PD-1 expression was more highly expressed in TAM than in splenic macrophages, and the expression level was correlated with the cholesterol content. Ectopic expression of the ABCA1/G1 ligand ApoA1 enhanced cholesterol efflux from TAM in the glioblastoma microenvironment and led to tumor clearance. The treatment of glioblastoma bearing mice with an engineered adenovirus expressing ApoA1 increased the phagocytic activity of TAM and decreased their PD-1 expression.

## OV delivery by monocytes/macrophages

As mentioned above, intravenous administration of OV still requires optimization. Indeed, most preclinical approaches and OV granted for clinical use are injected directly inside tumors. This is feasible for superficial tumors such as melanoma but is more complicated when cancer resides in deep organs. In theory, intravenous injection would enable these locations to be reached, as well as the different metastatic sites. However, neutralizing antibodies at baseline or induced after repeated OV administration is one of the main limiting factors for intravenous administration [1]. This limitation has been addressed by using liposomes [126] and exosomes [127] to mask the OV and to evade recognition by the immune system. An alternative approach is cell-mediated OV delivery, taking advantage of the natural migration patterns of monocytes to areas of tissue destruction, where they are actively recruited to the TME.

**Fig. 4:**
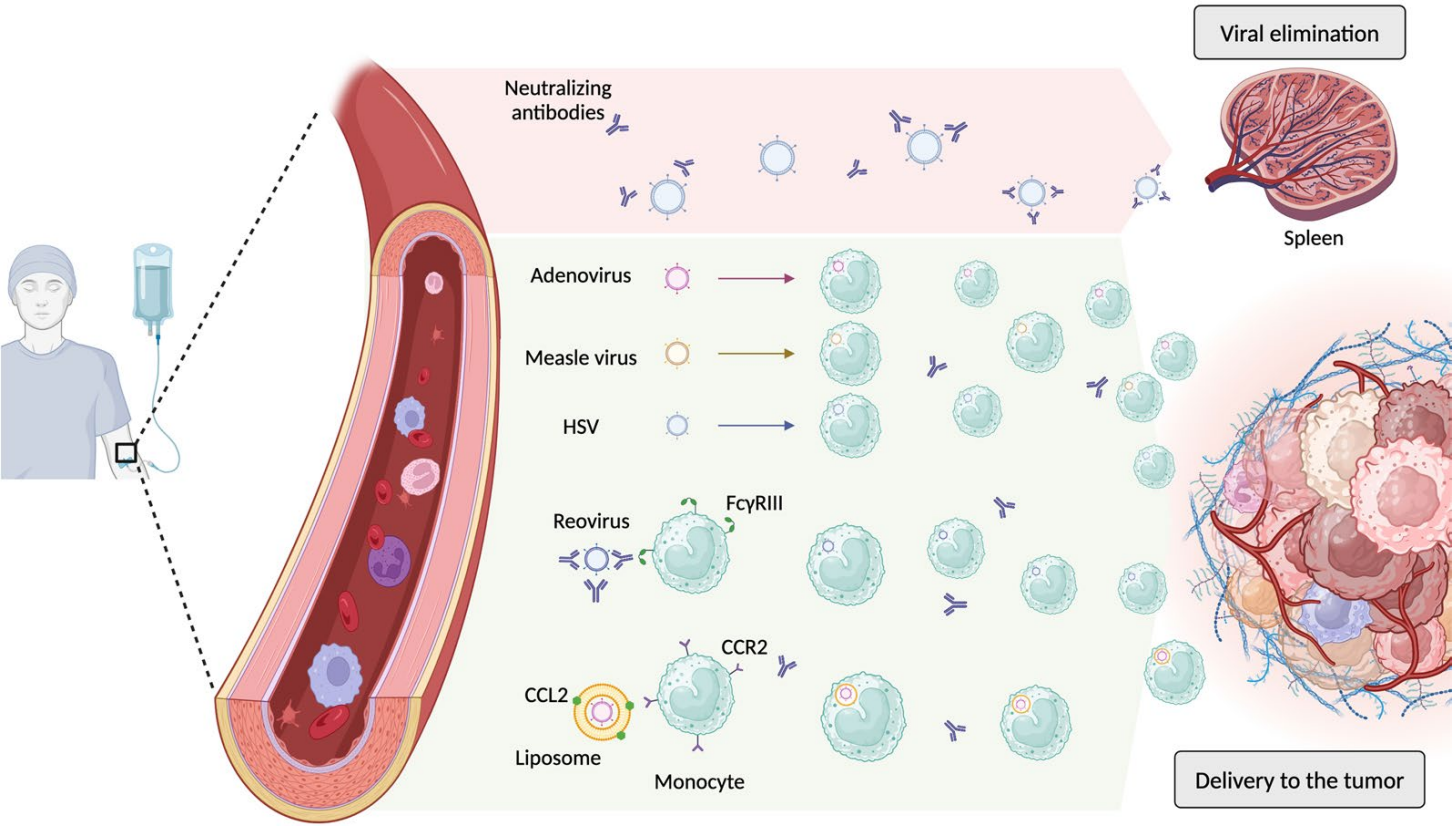
Oncolytic virus delivery to the tumor through intravenous administration. Free OVs in the plasma are prone to elimination by neutralizing antibodies through the reticulo-endothelial system (such as in the spleen). We further demonstrate the different strategies harnessing monocytes to deliver OV to tumors. Adenovirus, measle virus and HSV can be up taken by monocytes and transferred into the tumor. Monocytes can recognize reovirus coated with its neutralizing antibody through FcγRIII, incorporate it and migrate into the tumor. Finally, adenovirus coated in liposomes expressing CCL2 are recognized by circulating monocytes through CCR2, which leads to liposomes engulfment. All these strategies harness the monocyte capacity to migrate to areas of tissue destruction, facilitate delivery into tumors and protect OV from neutralizing antibodies

In preclinical models, intravenous injection of differentiated macrophages transduced with adenovirus increased viral infection of cancer cells in a xenograft prostate cancer model. More efficient tumor growth inhibition was achieved using this approach than in mice injected with free adenovirus [128]. Similar findings using monocytes were shown with an adenovirus in a xenograft pancreatic cancer model [129] and with a measles virus in an xenograft ovarian cancer model [130]. An alternative approach to exploiting monocytes as an OV delivery vector involved the encapsulation of an adenovirus into CCL2-coated liposomes; preferential uptake of encapsulated adenovirus by monocytes was confirmed in vitro. In vivo testing in an orthotopic xenograft prostate cancer model revealed enhanced therapeutic efficacy of encapsulated adenovirus rather than non-encapsulated adenovirus [131] (Fig. 4).

In human monocytes, Reale et al. showed that oHSV-1 can infect and replicate in monocytes from healthy donors. In co-culture, infected monocytes could transmit oHSV-1 to cancer cells. Interestingly, the capacity of monocytes to release oHSV-1 particles was increased when they were exposed to cancer cell conditioned media [132]. Regarding the impact on monocyte polarization, it was shown that infection with the Newcastle disease virus increased the secretion of the pro-inflammatory cytokines IFN, TNF, IL-6, and GM-CSF and the expression of the costimulatory molecule CD86 by healthy donor monocytes [133]. Such a pro-inflammatory phenotype would be beneficial for an antitumor response, but its induction in the periphery may decrease the bioavailability of the virus administered intravenously. Interestingly, in vitro polarized M2 macrophages were more easily infected than M1 macrophages.

As mentioned above, neutralizing antibodies are recognized as a detrimental factor for OV delivery to tumors after intravenous injection. Interestingly, however, for reoviruses, it appeared that neutralizing antibodies may facilitate their uptake by monocytes through FcγRIII. Loaded monocytes then transferred viral particles to cancer cells, which led to efficient cancer cell killing in vitro, while free antibody-reovirus complexes were unable to induce cancer cell death [134]. In humans, the administration of reovirus in ten patients with metastatic colorectal cancer showed preferentially transport in the blood and delivery to the tumor in mononuclear cells rather than as free viral particles [135]. This hitchhiking effect provided efficient protection against reovirus-specific neutralizing antibodies. Because of this, most clinical trials testing reovirus in cancer patients use an intravenous route of administration [136].

Taken together, these studies show that harnessing blood monocytes to deliver OV by intravenous infection is feasible. However, it is important to consider the impact of OV loading on monocyte pro-inflammatory status. Indeed, if monocytes become too pro-inflammatory, viral clearance can occur before reaching the tumor. Neutralizing antibodies decreases OV bioavailability even though, in the case of reoviruses, they also facilitate their uptake by monocytes. But neutralizing antibodies may also be a marker of a more robust immune response against infected cancer cells. In a phase I clinical trial with recurrent glioblastoma patients treated with HSV administered directly into the tumor, a positive serology against HSV was associated with better survival [137]. These patients also had a greater modification of T cell clonotype diversity and abundance, suggesting a more robust adaptive immune response. Therefore, while circulating anti-OV neutralizing antibodies are generally detrimental to OV delivery to the tumors, in some cases, they promote uptake by monocytes, such as for reovirus, and are associated with a better anti-tumoral immune response. In this context, the role of circulating monocytes in protecting and delivering OV into the tumor is of interest to optimize intravenous administration further.

## Perspectives and conclusion

TAM and OV interactions in tumors are complex. Several parameters of TAM are impacted, such as their activation status, infiltration, and phagocytic activity. While a pro-inflammatory phenotype of TAM is beneficial for antitumor immunity, it can be detrimental to OV efficacy. The evidence we have discussed with viral strains in different cancer models highlights the heterogeneity of these contexts. Indeed, the PRR involved in OV recognition differs according to the virus, notably for RNA and DNA viruses. In parallel, the level of baseline immune cell infiltration in the tumor could impact the response to virotherapy.

Some aspects of OV and TAM interactions still need to be explored. For instance, it has been described that some viruses promote angiogenesis [138] while others disturb it [139, 140]. How angiogenesis modification by OV impacts TAM recruitment and function can be of interest. The extracellular matrix has been shown to be able to restrict virus entry inside the tumors [141]. As TAM can cleave extracellular matrix containing collagen through metalloprotease secretion [14], this ability of TAM could be harnessed to improve OV penetrance in tumors.

Finally, data from the multiple studies with engineered OV that target specific anti-tumoral TAM functions are extremely promising. However, to make the interaction between TAM and OV more potent for anti-tumoral immunity, harnessing the differences between TAM anti-tumoral and antiviral responses will be necessary, ideally by keeping the former but preventing the latter. For example, we can speculate that virus recognition inhibition with PRR antagonization can be interesting. Solid preclinical evidence suggests that PRR activation is beneficial for anti-tumoral immunity [142], but as we have reviewed here, a rationale exists to inhibit them when in association with OV. We conclude that optimizing the interaction between OV and TAM may be pivotal and should be harnessed to deliver more potent ant-tumoral immunity. However, research is needed to unravel the intricate mechanisms underlying antitumor immunity and to pave the way towards more effective therapies.

## Data Availability

No datasets were generated or analysed during the current study.
